# Temporal multiomics gene expression data across human embryonic stem cell-derived polyhormonal cell differentiation

**DOI:** 10.1038/s41597-026-06606-8

**Published:** 2026-01-16

**Authors:** Abdurrahman Keskin, Hani J. Shayya, Achchhe Patel, Dario Sirabella, Barbara Corneo, Marko Jovanovic

**Affiliations:** 1 Department of Biological Sciences, Columbia University, New York, NY 10027 USA; 2 Mortimer B. Zuckerman Mind, Brain and Behavior Institute, Columbia University, New York, NY 10027 USA; 3 Stem Cell Core, Columbia University Irving Medical Center, New York, NY 10032 USA

**Keywords:** Transcriptomics, Proteomics, Stem-cell differentiation

## Abstract

Human embryonic stem cells (hESCs) provide a powerful *in vitro* model to study lineage specification and the regulatory programs underlying early human development. Here, we present a high-resolution, temporal multi-omics dataset tracking mRNA, translation, and protein expression dynamics during hESC differentiation into definitive endoderm and subsequent polyhormonal (PH) cells, a key pancreatic lineage. RNA-seq, ribosome profiling, and quantitative mass spectrometry-based proteomics were performed on matched samples collected at ten time points in biological duplicates, allowing detailed characterization of transcriptional, translational, and protein abundance changes over the differentiation timeline. The dataset exhibits high technical quality, with strong reproducibility between replicates and rigorous quality control metrics across all omics platforms. This extensive dataset provides critical insights into the complex regulatory mechanisms driving polyhormonal cell differentiation and serves as a valuable resource for the research community, enabling deeper exploration of mammalian development, endodermal lineage specification, and gene regulation.

## Background & Summary

Early developmental transitions involve rapid and highly structured changes in gene expression, yet the regulatory logic governing these shifts remains incompletely defined. Dissecting how gene regulatory layers interact over time requires experimental systems in which differentiation proceeds in a controlled and reproducible manner. Human embryonic stem cells (hESCs)^1^, which can be directed through stepwise lineage trajectories *in vitro*, offer such a system and enable high-resolution analysis of the molecular events that accompany early fate decisions.

**Fig. 1:**
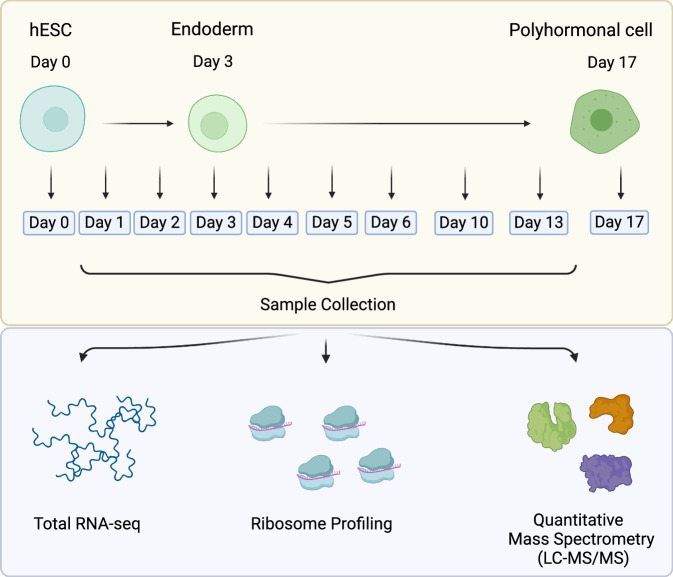
Experimental design and multi-omics data generation. hESCs were differentiated into the endoderm germ layer and subsequently into polyhormonal cells. Samples were collected at 10 different time points during the time-course of hESC differentiation, and matched samples were measured for RNA, translation, and protein levels by RNA-seq, ribosome profiling, and mass spectrometry, respectively. Created with BioRender.com.

To investigate the regulatory events that structure early differentiation, we generated a time-resolved multi-omics dataset capturing mRNA, translation, and protein dynamics across directed lineage trajectories. As part of a broader effort to profile all three embryonic germ layers, hESCs were differentiated into endoderm, mesoderm, and ectoderm lineages and further guided into polyhormonal pancreatic cells, cardiomyocytes, and motor neurons, respectively. The mesoderm-to-cardiomyocyte branch of this framework has been published previously^2^. In the present study, we focus on the endodermal branch and characterize the progression from pluripotent stem cells to polyhormonal pancreatic cells. At ten defined time points along this trajectory, we measured RNA levels by RNA sequencing (RNA-seq), translation level proxies by ribosome profiling (Ribo-seq), and protein abundance by quantitative mass spectrometry-based proteomics (LC-MS/MS), each performed in two biological replicates (Fig. 1). To our knowledge, the extensive temporal resolution and multi-omics design of this dataset make it one of the most comprehensive resources available for studying gene regulatory dynamics during human endoderm specification and pancreatic lineage development.

An integrated, time-resolved profiling strategy is essential for interpreting gene regulation during differentiation, as changes in transcript levels alone frequently fail to predict protein abundance^3–5^. Post-transcriptional mechanisms, including translational control and protein turnover, play major roles in shaping developmental transitions. Measuring mRNA, translation, and protein levels in parallel therefore provides a more complete view of regulatory architecture, enabling the dissection of how different layers of gene expression regulation contribute to lineage progression and when during differentiation they exert their strongest influence.

By resolving gene expression dynamics across mRNA, translational, and protein levels throughout endodermal differentiation, this dataset provides a valuable reference for dissecting regulatory mechanisms in human development. Its depth and temporal resolution make it well suited for comparative analyses, benchmarking of computational tools, and integrative modeling of gene expression dynamics. When viewed in the context of our broader effort to profile differentiation across all three germ layers, including the previously reported mesoderm-to-cardiomyocyte dataset^2^, this work contributes to an emerging cross-lineage framework for examining how regulatory strategies are deployed during early developmental transitions. We anticipate that this resource, both independently and when integrated with complementary datasets, will support a wide range of mechanistic and comparative studies, ultimately advancing our understanding of the molecular logic that governs early human development

## Methods

### Endoderm – polyhormonal pancreatic cell differentiation

Polyhormonal cell differentiation was performed as described previously^6^ with slight modifications. hESCs (RUES2 cell line) were maintained and expanded onto irradiated mouse embryonic fibroblasts (MEFs) (cat# GSC‐6001 G, ThermoFisher Scientific) plated onto gelatin-coated (cat# SF008, Millipore Sigma) plates with hES medium, constituted by DMEM/Ham’s F-12 supplemented with 20% KO-Serum Replacement, non-essential amino acids (1%), Penicillin-Streptomycin (1%), glutamine (1%), β-mercaptoethanol (55 μM) and FGF2 (10 ng/ml). Media was replaced every day. Cells were grown to confluency (80–90%) onto the irradiated MEFs before starting the differentiation. Irradiated MEFs were retained during the differentiation. On Day 0 (start of differentiation), the medium was changed to the differentiation medium composed of RPMI containing glutamine (1%), CHIR 99021 (2 μM), Activin A (100 ng/ml), and diluted MTG (0.45 mM). Cells were incubated in a humidified incubator at 37 °C in 5% CO_2_. On Day 1 and Day 2, the medium was changed to the differentiation medium composed of RPMI containing glutamine (1%), Activin A (100 ng/ml), diluted MTG (0.45 mM), FGF2 (5 ng/ml), and ascorbic acid (AA) (50 μg/ml). On Day 3, Day 4, and Day 5, the medium was changed to the differentiation medium composed of RPMI containing glutamine (1%), diluted MTG (0.45 mM), dorsomorphin (0.75 μM), FGF10 (50 ng/ml), and B27 (1%). On Day 6, Day 7, and Day 8, the medium was changed to the differentiation medium composed of DMEM high glucose (HG) containing glutamine (1%), AA (50 μg/ml), FGF10 (50 ng/ml), B27 (1%), SANT-1 (0.25 μM), Retinoic Acid (RA) (2 μM), and NOGGIN (50 ng/ml). On Day 9 and Day 11, the medium was changed to the differentiation medium composed of DMEM high glucose (HG) containing glutamine (1%), AA (50 μg/ml), B27 (1%), NOGGIN (50 ng/ml), and SB 431542 (6 μM). On Day 13, the medium was changed to the differentiation medium composed of SFD medium containing glutamine (1%), AA (50 μg/ml), NOGGIN (50 ng/ml), SB 431542 (6 μM), L-658548 (1 μM), and D-Glucose (20 mM). After Day 13, the same medium was replaced every 3 days.

### Sample collection and preparation

Sample collection was performed on Day 0, Day 1, Day 2, Day 3, Day 4, Day 5, Day 6, Day 10, Day 13, and Day 17, for a total of ten time points. For each time point, cells were harvested from one entire six-well plate at ≥90% confluency, corresponding to approximately 18 million cells in total. One well (~3 million cells) was used for proteomics analysis. The remaining five wells (~15 million cells) were collected for RNA-seq and ribosome profiling. From this material, approximately one-tenth (~1.5 million cells) was allocated for RNA extraction and RNA-seq, while the remaining ~13.5 million cells were used for ribosome profiling.

For RNA-seq and ribosome profiling, plates were placed on ice and cold polysome lysis buffer (20 mM Tris pH 7.4, 250 mM NaCl, 15 mM MgCl₂, 100 µg/ml cycloheximide, 1 mM dithiothreitol (DTT), and 0.5% Triton X-100, supplemented with 10 × protease inhibitor, 0.04 U/µl TurboDNase, and 0.4 U/µl RNasin) was added to each well. Cells were scraped directly into the cold buffer and mixed several times by pipetting, followed by homogenization through three passes on a 23-gauge needle. All steps were performed on ice to minimize RNA degradation and preserve ribosome–mRNA interactions. Cells were not pretreated with cycloheximide.

For proteomics analysis, cells were lysed in ice cold urea buffer (8 M urea, 75 mM NaCl, 50 mM Tris pH 8.0, 1 mM EDTA). After buffer addition, cells were mixed several times by pipetting and then were continuously rotated on the tube rotator for 20 min at room temperature to promote efficient lysis.

### Cell line authentication and quality control

We utilized the RUES2 hESC line, which was derived at Rockefeller University. Detailed information regarding the derivation and characterization of this cell line can be found through the following link: https://xenopus.rockefeller.edu/stemcell/rues2. Additional characterization data are available on the hPSCreg website, where this cell line is registered: https://hpscreg.eu/cell-line/RUESe002-A.

The RUES2 cell line has been authenticated and tested for integrity at Columbia Stem Cell Core, as well. Karyotyping was performed at passages 18, 34, and 39, confirming a normal human female karyotype. Furthermore, mycoplasma contamination testing was conducted multiple times during the cell culture process, with all results indicating no mycoplasma contamination.

### RNA-seq library construction

Homogenates were cleared by centrifugation at 13,000 × g for 10 min at 4 °C. Total RNA was isolated from the collected supernatant by QIAGEN RNeasy kit, and RNA integrity was assessed with a Bioanalyzer (Agilent). All samples exhibited high RNA quality, with RIN values greater than 9. For each library, 100 ng of total RNA was used as input. Ribosomal RNA was depleted by the NEBNext rRNA depletion kit (Human/Mouse/Rat) according to the manufacturer’s instructions. Strand-specific sequencing libraries were generated from rRNA-depleted total RNA samples by using NEBNext Ultra II Directional RNA Library Prep Kit for Illumina following the manufacturer’s instructions. The libraries were quantified using a Qubit dsDNA HS kit (Thermo Fisher Scientific), and the library quality was assessed by a Bioanalyzer (Agilent). Paired-end sequencing was performed using an Illumina NextSeq 500 desktop sequencer with a read length of 75 bases.

### Ligation-free ribosome profiling

Ribosome profiling was performed as described previously^7,8^ with slight modifications. Briefly, homogenates were clarified by centrifugation at 13,000 × g for 10 min at 4 °C. A total RNA amount of 1.5–5 μg per sample, contained in ~400 μL of polysome lysis buffer, was used for nuclease digestion. Clarified cell lysates were treated with *E. coli* RNase I (Ambion) and incubated for 30 min at room temperature to generate ribosome-protected fragments (RPFs). Following digestion, the lysate was gently layered on top of 2 mL of 50% sucrose solution, and RPFs were pelleted using an ultracentrifuge (Beckman, TLA-100.3 rotor) at 70,000 rpm for 3.5 hours at 4 °C.

RPF pellets were resuspended in 1 mL TRIzol, and phase separation was performed by adding 200 μL chloroform, shaking briefly, and centrifuging at 12,000 × g for 15 min at 4 °C. The aqueous phase was recovered, mixed with GlycoBlue and isopropanol, and precipitated overnight at −80 °C. Pellets were collected by centrifugation at 12,000 × g for 10 min at 4 °C, washed with 75% ethanol, air-dried, and resuspended in 10 μL nuclease-free water.

For size selection, ~5 μL of recovered RPFs were resolved on a 15% polyacrylamide TBE–urea denaturing gel, run alongside synthetic RNA size markers corresponding to 26 nt (lower) and 34 nt (upper)^8^. RNA fragments between 28–34 nt were excised and eluted. Size-selected fragments were then dephosphorylated with T4 Polynucleotide Kinase (NEB).

After dephosphorylation, RPFs were quantified using a Bioanalyzer Small RNA kit (Agilent). Based on this measurement, 15 ng of dephosphorylated RPFs were used as input for ligation-free library preparation using the SMARTer Small RNA-Seq Library Preparation Kit (Takara Bio), performed according to the manufacturer’s instructions^9^.

Following library generation, rRNA depletion was performed using a biotinylated oligo pool targeting human rRNAs^8^. Briefly, libraries were denatured with the oligo pool at 100 °C for 90 seconds, annealed by slow cooling to 37 °C, and then incubated at 37 °C for an additional 15 min before binding to pre-washed MyOne Streptavidin C1 Dynabeads. The depleted libraries (~10 μL) were recovered after bead removal.

Depleted libraries were PCR-amplified for 12 cycles using the reagents included in the SMARTer kit. Final libraries were quantified using a Qubit dsDNA HS kit (Thermo Fisher Scientific) and evaluated for size distribution and purity using a Bioanalyzer High Sensitivity DNA kit (Agilent). All libraries exhibited the expected size profiles and adequate concentration and were therefore included for sequencing on an Illumina NextSeq 500 with a read length of 50 bases.

### Liquid Chromatography-Tandem Mass Spectrometry (LC-MS/MS)

Cell lysates prepared in urea buffer were cleared by centrifugation at maximum speed (~20,000 × g) for 15 min at room temperature. Protein concentrations were measured using the Pierce™ BCA protein assay kit (Thermo Fisher Scientific), and all samples exhibited concentrations greater than 1 µg/µL. For each sample, 20 µg of total protein in 20 µL of urea buffer were taken forward for reduction and alkylation.

Proteins were reduced to a final concentration of 5 mM dithiothreitol (DTT). DTT was supplied as a 500 mM stock, diluted to a 50 mM working stock, and added at around 10% of the sample volume. Samples were incubated for 45 min at 25 °C with gentle shaking. Reduced proteins were alkylated with iodoacetamide (IAA) at a final concentration of 10 mM. IAA was supplied as a 500 mM stock, diluted to a 100 mM working stock, and added at around 10% of the sample volume. Alkylation proceeded for 45 min at 25 °C with gentle shaking in the dark.

Following alkylation, samples were diluted 1:5 with 50 mM Tris-HCl (pH 8.0) to reduce the urea concentration below 2 M. Proteins were digested overnight (~16 h) at 25 °C with sequencing-grade trypsin (Promega, cat. V5113, 0.5 µg/µL) at an enzyme-to-substrate ratio of 1:50 (w/w), with gentle shaking. After digestion, samples were acidified to 1% (v/v) final formic acid (FA) using a 10% (v/v) FA stock to enable efficient binding to C18 material.

Peptide mixtures were desalted using in-house packed C18 StageTips containing two C18 discs in a 200 µL pipette tip. StageTips were conditioned sequentially with 100 µL methanol, 100 µL 80% acetonitrile / 0.2% FA, and 2 × 100 µL 0.2% FA, with centrifugation at ~3,500 × g for 2 min between steps. Acidified peptide samples were loaded onto the conditioned StageTips and centrifuged at ~3,500 × g for 2 min. Columns were then washed twice with 100 µL 0.2% FA. Peptides were eluted with 60 µL of 60% acetonitrile / 0.2% FA^10^. The eluates were dried in a vacuum concentrator at 45 °C with a vacuum pressure of approximately 5.1 mbar (~1 hour). Dried peptides were reconstituted in 20 µL of 3% acetonitrile / 0.2% FA for the subsequent liquid chromatography-tandem mass spectrometry (LC-MS/MS) analysis.

LC-MS/MS analysis was performed on a Q-Exactive HF. Approximately 0.5 µg of total peptides (corresponding to 5 µL of the reconstituted solution) were analyzed on a Waters M-Class UPLC using an IonOpticks Aurora ultimate column (1.7 μm, 75 μm × 25 cm) coupled to a benchtop ThermoFisher Scientific Orbitrap Q Exactive HF mass spectrometer. Peptides were separated at a flow rate of 400 nL/min with a linear 95 min gradient from 2% to 22% solvent B (99.9% acetonitrile / 0.1% FA), followed by a linear 20 min gradient from 22 to 30% solvent B, followed by a linear 9 min gradient from 30% to 60% solvent B, followed by 36 min of wash and column equilibration in 98% solvent A (99.9% water / 0.1% FA) and 2% solvent B. Each sample was run for 160 min, including sample loading and column equilibration times. The samples were measured in a Data Independent Acquisition (DIA) mode. MS1 Spectra were measured with a resolution of 120,000, an AGC target of 5e6 and a mass range from 350 to 1650 m/z. 47 isolation windows of 28 m/z were measured at a resolution of 30,000, an AGC target of 3e6, normalized collision energies of 22.5, 25, 27.5, and a fixed first mass of 200 m/z.

### Data analysis

#### RNA-seq and ribosome profiling

##### Annotation

A BED file containing all transcripts in the hg38 human reference genome was downloaded from Ensembl (version 32). The transcript set was then filtered using a custom Python script, which was built predominantly using tools from the plastid package (v0.6.1). Only transcripts with an annotated coding sequence were retained. Duplicates and transcripts from unlocalized contigs were excluded from subsequent analysis. Transcript to gene name mappings were compiled from Ensembl using custom R code built using the biomaRt package (v2.62.0). For RNA-seq alignment using STAR^11^, the final transcript set was exported as a GTF2 file using a custom Python script and used to construct a STAR index using the genomeGenerate command and specifying --sjdbOverhang 74. For Ribo-seq alignments, a separate STAR index was constructed using the same GTF2 file but specifying --sjdbOverhang 29. The GTF2 file was also used to construct the metagene annotation files used by programs in the plastid package. This was accomplished using the plastid metagene generate program and the flags --landmark cds_start and --annotation_format GTF2.

##### Read processing and alignment

RNA-seq reads were aligned to the genome using STAR. Unprocessed reads were fed to STAR using the --runMode alignReads --outSAMtype BAM SortedByCoordinate --outFilterType BySJout –outFilterIntronMotifs RemoveNoncanonicalUnannotated --outSAMstrandField intronMotif and --outFilterMultimapNmax 10 flags. Downstream tools to combine RNA-seq and Ribo-seq data were built to use single-end data, so the sense read of every aligned pair was extracted using samtools (v1.19) view (-b -q 30 -f 129), and the resulting bam file was used for downstream analysis.

For Ribo-seq, reads were pre-processed using cutadapt (v1.17) to remove the first three nucleotides and the poly-A tail (--cut 3 -a “A{15}” -j 8 --nextseq-trim = 20 --minimum-length 17). Bowtie (v1.2.2) was used to align reads to the human 45S pre-rRNA (NR_146144.1, downloaded from GenBank) using the -v 0 and --un flags to remove rRNA reads from the analysis. Reads were aligned to the genome using STAR (--runMode alignReads–outSAMtype BAM SortedByCoordinate –alignSJoverhangMin 400 –outFilterMismatchNmax 0 --outFilterMatchNmin 15 --outFilterMultimapNmax 1--quantMode TranscriptomeSAM). Post-processing was performed using tools from the plastid package. The plastid peptidyl-site (P-site) program was used to estimate the location of ribosomal P-sites for each read length in each library (--min_counts 50 --require_upstream --min_length 17 --max_length 35). P offsets were generally in the range of 6–13, and some manual corrections were necessary in cases where the P-site program identified an incorrect peak. All manual corrections were done blinded to sample library identity. A default of 13 was used in cases where the P-site could not be clearly determined. For all subsequent analyses, reads were reduced to single counts offset from the 5′ end of the read by the value computed by the P-site program.

##### Read counting

Read counting of the RNA-seq and Ribo-seq data was performed using genomic alignments and a custom Python script built using tools from the plastid package. Briefly, for each gene, all associated transcripts were combined to create a single “meta-transcript”. Any genomic position mapping to the CDS of any annotated transcript was assigned to the CDS of the meta-transcript. Conversely, the 5′- and 3′UTRs of the meta-transcript were comprised of genomic positions assigned to the 5′/3′UTR of at least one transcript but never to a CDS. Ribo-seq reads (reduced to a single count with a 5′ offset as defined above) in each defined meta region (5′UTR, CDS, 3′UTR) were counted. RNA-seq reads (reduced to a single count at the 5′ end) were counted in the meta-CDS and for the full meta-transcript. Count matrices were read into R and CDS counts for RNA-seq, and Ribo-seq were propagated forward into DESeq2^12^ (v1.46.0) using the DESeqDataSetFromMatrix function. CDS counts from genome-aligned libraries were normalized using DESeq2’s median of ratios method.

#### Gene body coverage and sequencing depth saturation analysis

Gene body coverage profiles were assessed using RSeQC (v5.0.4). The *geneBody_coverage.py* module was run on STAR-aligned BAM files using a custom BED12 transcript annotation generated from the Ensembl GRCh38.104 GTF. For each library, RSeQC computed normalized read density across the 5′–3′ axis of annotated transcripts, allowing evaluation of positional bias and uniformity of transcript coverage.

Sequencing depth saturation analysis was conducted using read count files generated from down-sampled BAM alignments. For each RNA-seq BAM file, mapped reads were downsampled using samtools view -s in 5-million–fragment increments, starting at 5 M and extending up to the full mapped-read depth of each individual sample. Gene-level quantification at each subsampled depth was performed using featureCounts (v2.1.1) with exonic counting (-t exon -g gene_id). For each depth, we calculated the number of genes detected with ≥10 counts. Detection tables were aggregated and visualized in R (v4.4.0) using ggplot2 (v3.5.1) to generate sample-wise saturation curves.

#### Proteomics: Search and quantification

Proteomics raw data were analyzed using the library-based DIA search method (utilizing both DDA- and direct DIA-generated libraries) on SpectroNaut (v20.0) using a human UniProt database (Homo sapiens, UP000005640), under BSG factory settings, with automatic cross-run median normalization and global imputation. Protein group data were exported for subsequent analysis.

#### GO term enrichment analysis

Gene ontology enrichment analysis was conducted using the WebGestalt^13^ (http://www.webgestalt.org, v2024) platform using the Gene Set Enrichment Analysis (GSEA)^14^ on biological processes non redundant terms.

#### Principal component analysis and batch correction

To account for batch effects between biological replicates, we applied the removeBatchEffect function from the limma^15^ R package (v3.60.6). Batch-corrected, log2-transformed expression data were then used to perform principal component analysis (PCA) using the prcomp() function in R (v4.4.0). PCA plots were generated based on the first two principal components to visualize sample clustering and temporal trajectories.

## Data Records

RNA-seq and ribosome profiling datasets have been deposited in the NCBI GEO database under accession numbers GSE305933^16^ and GSE305934^17^, respectively. The mass spectrometry data are available in the MassIVE repository with the accession number MSV000098914^18^.

The RNA-seq, ribosome profiling, and proteomics datasets include both raw data and processed quantification files, deposited in the repositories mentioned above. The raw RNA-seq and ribosome profiling files are labeled to indicate the data type, biological replicate, and time point of collection during polyhormonal cell differentiation. Processed gene-level count matrices are provided for both RNA-seq and ribosome profiling, in normalized and raw formats. Columns in these tables represent samples from two biological replicates collected across multiple time points, with column names reflecting replicate and day identifiers. The mass spectrometry dataset includes raw LC-MS/MS data acquired in DIA mode, similarly labeled by replicate and time point. Protein group quantification tables are included with and without imputation, containing intensity values for identified protein groups across time-course samples. All quantification files are organized in a wide format, with rows representing genes or proteins and columns corresponding to replicate-day combinations.

In addition to these primary datasets, GEO accessions GSE305933^16^ and GSE305934^17^ also include the BED12 reference file used for gene-body coverage analysis (geneBodyCoverage_reference_3500.bed12) as well as the full differential-expression result tables for RNA-seq (RNAseq_fullDiffExpressionResults_polyhormonal_vs_hESC.xlsx) and ribosome profiling (Riboseq_fullDiffExpressionResults_polyhormonal_vs_hESC.xlsx**)**. These tables summarize log₂ fold changes, p-values, and adjusted p-values (q-values) for the comparison between polyhormonal cells and hESCs. The full differential-expression results for the proteomics dataset (Proteomics_fullDiffExpressionResults_polyhormonal_vs_hESC.xlsx) have been deposited together with the proteomics data in MassIVE under accession MSV000098914^18^. This table likewise reports log₂ fold changes, p-values, and q-values for the polyhormonal versus hESC comparison at the protein-group level.

We additionally provide an annotation file through Figshare^19^ (hg38_Ensembl_v32_coding_transcripts.bed), the BED file containing Ensembl v32 annotated coding transcripts used for various downstream analyses.

## Technical Validation

### Validation of biological consistency

**Fig. 2:**
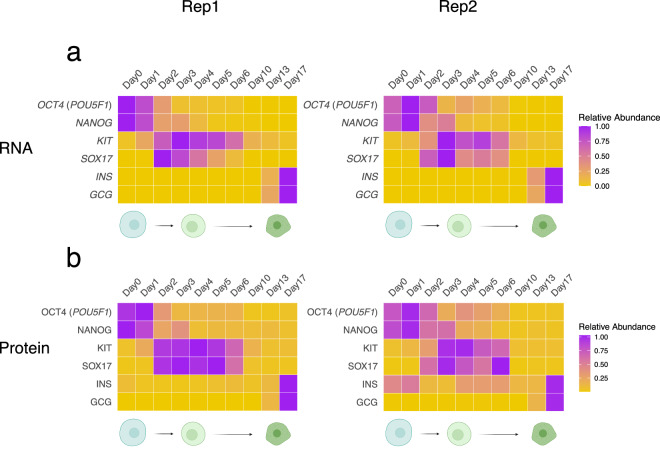
Expression of differentiation markers at (**a**) RNA and (**b**) protein levels during hESC to polyhormonal cell differentiation for both replicates. *OCT4* (*POU5F1*) and *NANOG*, stemness markers; *KIT* and *SOX17*, endoderm markers; *INS* and *GCG*, polyhormonal cell markers. The color scale represents normalized expression values (expression levels normalized to the maximum expression value of each gene across all time points), with purple indicating high expression and yellow indicating low expression.

**Fig. 3:**
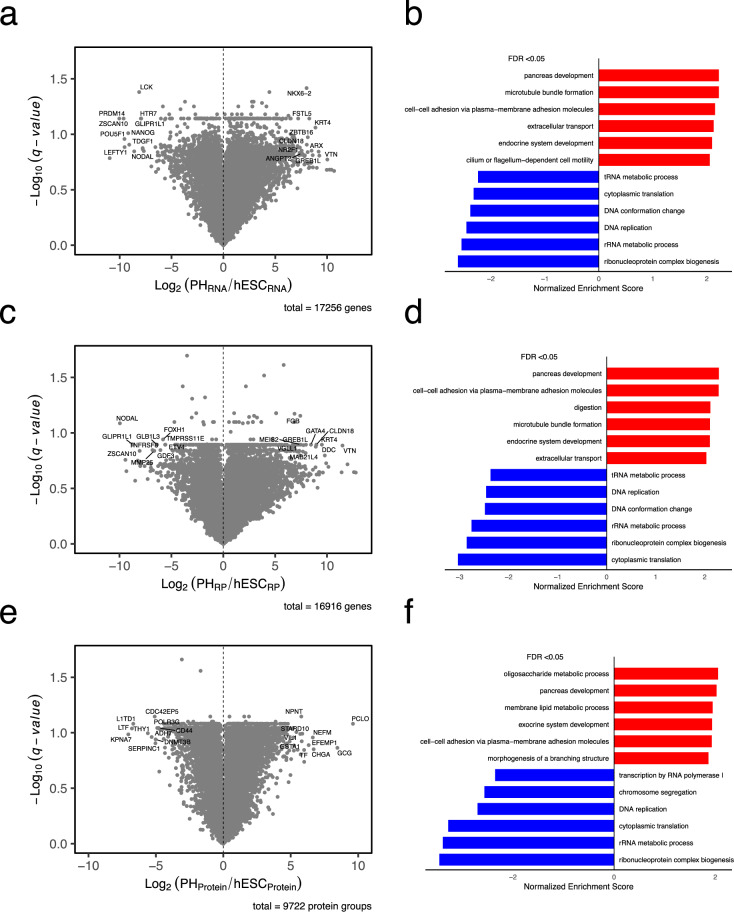
Comparison of polyhormonal cells to undifferentiated hESC expression at RNA, translation, and protein levels. (**a,c,e**) Volcano plots comparing gene expression between polyhormonal cells and undifferentiated hESCs at the (**a**) RNA, (**c**) translation, and (**e**) protein levels, respectively. The x-axis represents the log₂ fold change of gene expression (PH/hESC), and the y-axis represents the adjusted p-value (q-value), calculated using two-tailed Student’s t-tests followed by Benjamini–Hochberg correction. All genes are shown in gray, and the top ten genes with the strongest increase and decrease in fold change that meet the q-value cutoff (q < 0.15) are labeled with their gene IDs. PH = Polyhormonal cell, RP = Ribosome profiling. (**b,d,f**) Gene Ontology (GO) analysis performed using GSEA with all genes ranked by their fold change at the (**b**) RNA, (**d**) translation, and (**f**) protein levels revealed enrichment of GO terms related to pancreatic development, cell–cell adhesion, and endocrine system development, and depletion of terms linked to DNA replication, cytoplasmic translation, and ribonucleoprotein complex biogenesis. GO terms shown passed an False Discovery Rate (FDR) threshold of <0.05.

We evaluated the expression of stage-specific markers at both the mRNA and protein levels to confirm the progression of differentiation. Undifferentiated hESCs showed high expression of the stemness markers *OCT4* (*POU5F1*) and *NANOG*. Transition to definitive endoderm was supported by increased expression of *KIT* and *SOX17*. At the final stage of this differentiation protocol, cells acquired characteristics of polyhormonal cells, a pancreatic lineage capable of differentiating into glucagon-producing cells and defined by the concurrent expression of insulin (*INS*) and glucagon (*GCG*). Both markers were detected at the transcript and protein levels, confirming successful induction of this lineage (Fig. 2a,b). A slight delay in the activation of the endoderm markers *KIT* and *SOX17* was observed in replicate 2 compared to replicate 1, consistent with minor differences in differentiation kinetics. This modest temporal offset reflects expected biological variability rather than technical variation and does not affect the overall differentiation trajectory or interpretation of lineage progression. Comparative analysis of polyhormonal cells and hESCs using RNA-seq, Ribo-seq, and proteomics identified distinct sets of differentially expressed genes and proteins (Fig. 3a,c,e). Gene Ontology (GO) analysis, performed using Gene Set Enrichment Analysis (GSEA) with all genes ranked by their fold change, revealed significant enrichment of processes related to pancreas development, endocrine system development, and extracellular organization, and depletion of processes associated with DNA replication, ribosome biogenesis, and cytoplasmic translation (Fig. 3b,d,f).

Differentiation efficiency was not quantitatively assessed in this study, and we therefore did not determine the proportion of cells expressing lineage-specific markers at each stage. This represents a limitation of the dataset and should be considered when interpreting potential variability across time points. Although the differentiation was performed on irradiated MEF feeders, their contribution to the molecular profiles is expected to be minimal: at each time point, a single well of a six-well plate contained approximately three million hESC-derived cells at ≥90% confluency, together with ~300,000 non-dividing irradiated MEFs, corresponding to less than one-tenth of the total cell population. Moreover, all transcriptomics, translatomics, and proteomics analyses were restricted to the human reference database, further minimizing the impact of MEFs on downstream results. While the absence of quantitative efficiency measurements is a limitation, the strong and coherent induction of stage-appropriate markers at both the RNA and protein levels supports the successful progression of differentiation in this dataset.

### Validation of dataset reproducibility

**Fig. 4:**
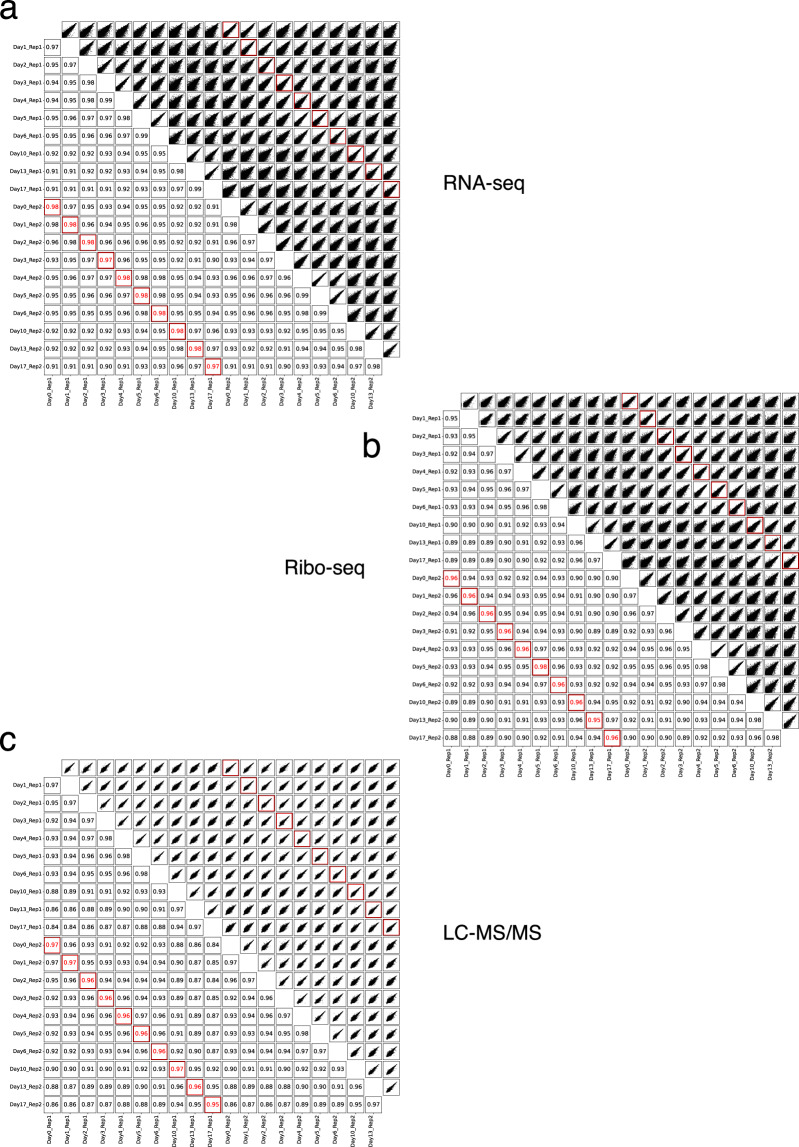
Correlation analysis of samples across all time points and replicates for each omics dataset: (**a**) RNA-seq, (**b**) Ribo-seq, and (**c**) LC–MS/MS. Each panel displays pairwise comparisons between samples, with the upper right triangle showing scatter plots and the lower left triangle indicating Pearson correlation coefficients (R). Biological replicate pairs from the same time point are outlined in red, with both their scatter plots (upper right) and correlation values (lower left) highlighted.

To assess reproducibility and capture temporal relationships within the dataset, we calculated pairwise Pearson correlation coefficients for all samples from the RNA-seq, Ribo-seq, and proteomics (LC-MS/MS) experiments (Fig. 4a–c). While we acknowledge that the use of two biological replicates per time point may limit statistical power and increase sensitivity to biological variability, this design reflects a balance between extensive temporal coverage across ten differentiation stages and the inclusion of three complementary omics layers. Importantly, the strong concordance between replicates across RNA-seq, Ribo-seq, and proteomics, together with the clear and gradual molecular transitions observed across time points, provides confidence that the dataset robustly captures dynamic changes during differentiation.

**Fig. 5:**
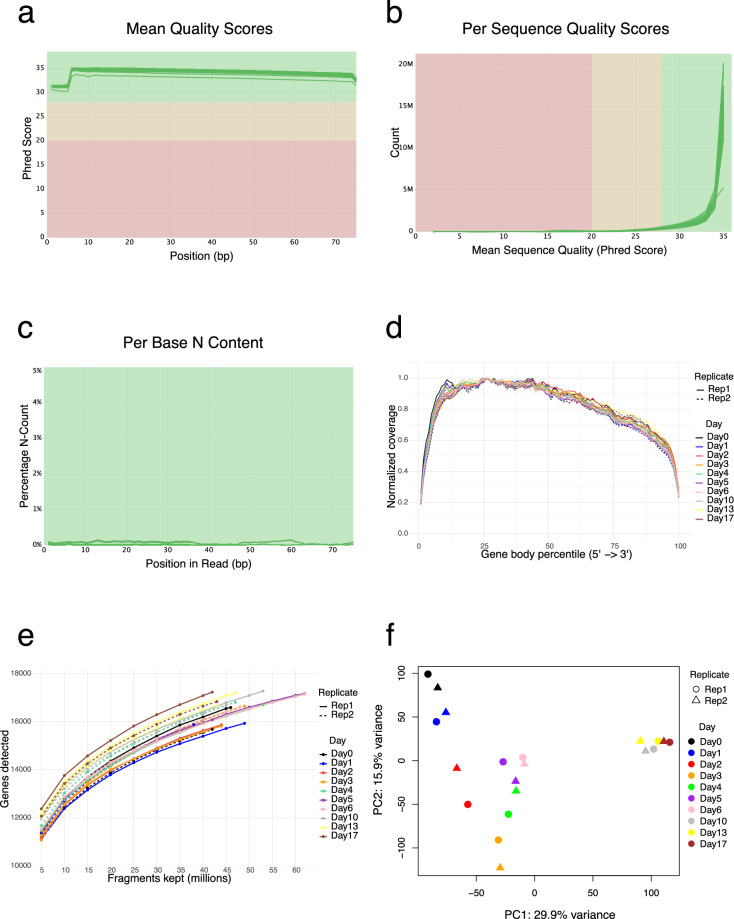
Quality control and PCA of the RNA-seq data. (**a**) Mean base quality scores across read positions, shown as Phred scores for all libraries. (**b**) Distribution of mean quality scores across all reads in each sample. (**c**) Percentage of undetermined bases (N) at each position along the reads, with individual samples represented by green lines. Shaded regions indicate quality thresholds: green for high quality, yellow for moderate quality, and red for low quality. (**d**) Gene-body coverage profiles showing normalized read coverage from the 5′ to 3′ end across transcripts, indicating uniform coverage and minimal positional bias. (**e**) Sequencing-depth saturation curves showing the number of genes detected (≥10 counts) as a function of the number of reads retained, demonstrating adequate sensitivity at the achieved read depth. (**f**) Principal Component Analysis (PCA) showing variance in RNA expression profiles across the differentiation time course, with separation by day and clustering of biological replicates.

**Fig. 6:**
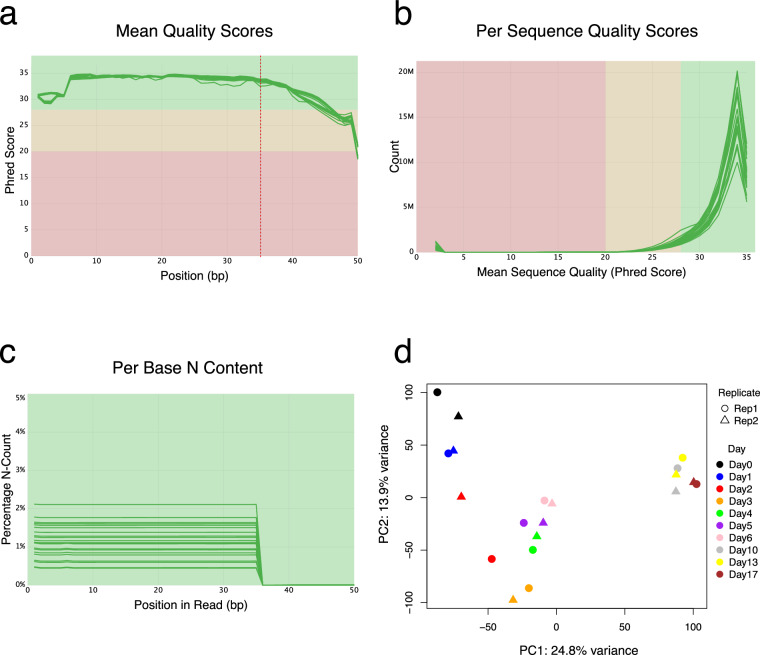
Quality control and PCA of the Ribo-seq data. (**a**) Mean base quality scores across read positions, shown as Phred scores for all libraries. (**b**) Distribution of mean quality scores across all reads in each sample. (**c**) Percentage of undetermined bases (N) at each position along the reads, with individual samples represented by green lines. Shaded regions indicate quality thresholds: green for high quality, yellow for moderate quality, and red for low quality. (**d**) Principal Component Analysis (PCA) showing variance in translation profiles across the differentiation time course, with separation by day and clustering of biological replicates.

Replicates from the same time point demonstrated consistently high agreement across all data types, with correlation coefficients of 0.97–0.98 for RNA-seq, 0.95–0.98 for Ribo-seq, and 0.95–0.97 for proteomics. Strong correlations were also observed between adjacent time points within and across replicates, reflecting the gradual and coordinated shifts in gene expression that occur during differentiation. Notably, some correlations between different time points, particularly among the later stages (days 10, 13, and 17), were nearly as high as those between replicates of the same day. This pattern likely reflects the close transcriptional, translational, and proteome similarity of cells at these terminal stages of differentiation, which is consistent with the PCA results showing that samples from these time points cluster closely together and are well separated from earlier stages (Figs. 5f, 6d, 8c).

### QC on the RNA-seq data

Quality assessment of all RNA-seq libraries was carried out using FastQC^20^ (v0.12.0), with sequencing depth ranging from 20 to 30 million paired-end reads per sample and approximately 40% unique reads. Summary reports were then compiled with MultiQC^21^ (v1.22.3), confirming high data quality across all libraries, as indicated by metrics such as mean base quality scores (Fig. 5a), per-sequence quality scores (Fig. 5b), and per-base N content (Fig. 5c).

To further evaluate library quality and sequencing coverage, we performed gene body coverage and saturation analyses. Gene-body coverage profiles were uniform across the 5′–3′ axis for all samples, indicating consistent transcript representation and minimal positional bias (Fig. 5d). The depth–sensitivity curves, calculated using a detection threshold of ≥10 counts per gene, showed that gene detection approached saturation as sequencing depth increased, with diminishing gains in newly detected genes at higher read counts. Both replicates followed highly similar trajectories across all time points, confirming consistent coverage and sufficient sequencing depth for comprehensive transcript quantification (Fig. 5e).

Following alignment and quantification, Principal Component Analysis (PCA) was performed to visualize variation in gene expression profiles across the differentiation time course (Fig. 5f). The variance is clearly captured across the differentiation timeline, with replicates grouping closely together and samples following a distinct time-dependent trajectory. This separation along the principal components highlights the dynamic transcriptional changes that occur as hESCs progress toward polyhormonal cell differentiation.

### QC on the Ribo-seq data

The quality of raw Ribo-seq reads was assessed using FastQC. Sequencing depth ranged from 40 to 70 million single-end reads per sample, with approximately 25% unique reads. A gradual decline in mean quality scores toward the end of the 50 bp ribosome footprints was observed, consistent with the presence of the poly(A) tail introduced during library preparation for cDNA synthesis (Fig. 6a). Other quality metrics, including per-sequence quality scores and per-base N content, indicated consistently high-quality data across samples (Fig. 6b,c). PCA of the Ribo-seq data showed that replicates from the same day clustered closely together, with clear separation of samples by time point, consistent with the trends observed in the RNA-seq data (Fig. 6d).

**Fig. 7:**
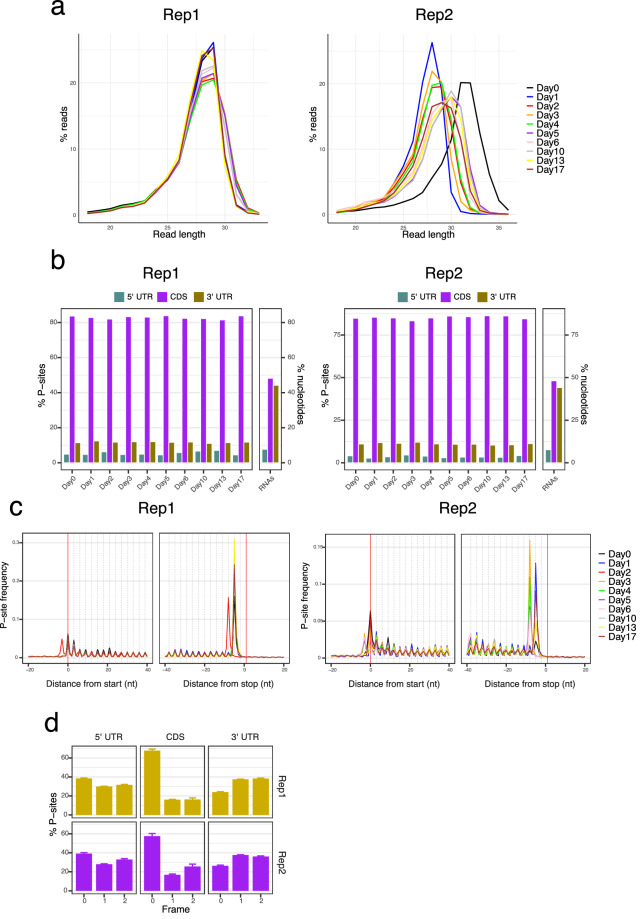
Diagnostic analysis of the Ribo-seq data. (**a**) Read length distribution of ribosome-protected fragments across the differentiation time course for replicate 1 and replicate 2. (**b**) Distribution of peptidyl-sites (P-sites) within transcript regions (5′ UTR, CDS, and 3′ UTR) for each day of differentiation in Rep1 and Rep2. The bar labeled “RNAs” represents the expected distribution from random RNA fragmentation. (**c**) P-site frequency relative to the start and stop codons across all time points in Rep1 and Rep2. (**d**) Distribution of P-sites among the three nucleotide frames (0, 1, and 2) within the 5′ UTR, CDS, and 3′ UTR regions for Rep1 and Rep2, averaged across all time points.

To validate that the ribosome profiling data were both technically sound and biologically representative, we carried out diagnostic analyses with riboWaltz^22^ (v2.0). Read length distributions revealed a predominant enrichment of ribosome-protected fragments at the expected 28–30 nucleotide size in both replicates (Fig. 7a). The highest proportion of reads mapped to coding sequences (CDS), reflecting active translation (Fig. 7b). A key feature of ribosome profiling data is the trinucleotide periodicity of ribosome footprints along coding sequences, arising from the ribosome advancing one codon at a time during translation. Consistent with this, P-site profiles exhibited clear codon-level periodicity along the CDS in both replicates (Fig. 7c). Frame distribution analysis further confirmed a strong enrichment of P-sites in frame 0 within CDS regions, with a more balanced distribution in the 5′ and 3′ UTRs (Fig. 7d). Together, these features confirm that the Ribo-seq data are of high quality and capture the expected biological patterns.

### QC on the LC-MS/MS data

**Fig. 8:**
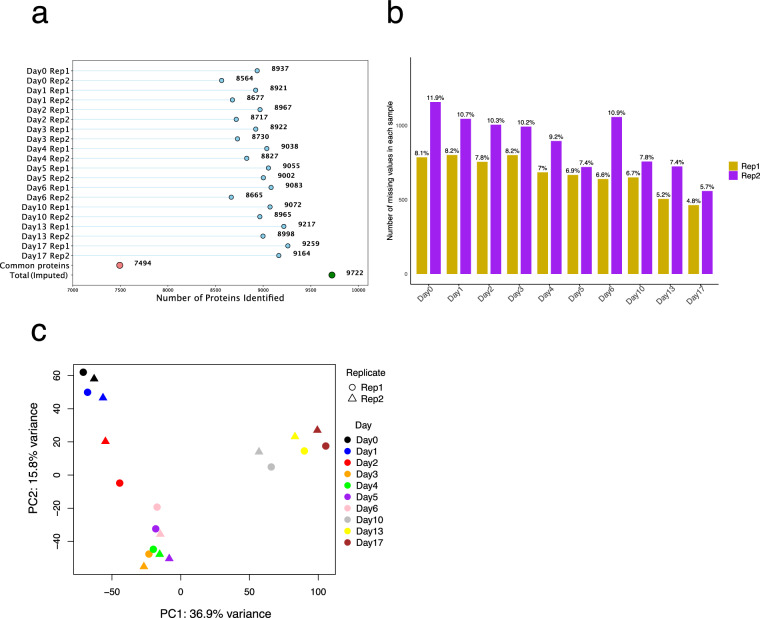
Quality control and PCA of the LC-MS/MS data. (**a**) Number of protein groups identified across the differentiation time course for replicate 1 and replicate 2. Blue dots indicate the number of protein groups identified in each sample without imputation. The red dot marks the 7,494 protein groups consistently detected in all samples, and the green dot marks the total number of protein groups identified with imputation (9,722). (**b**) Percentage and number of missing values in protein group identifications across the time course for Rep1 and Rep2. (**c**) Principal Component Analysis (PCA) showing variance in protein expression profiles across the differentiation time course, with separation by day and clustering of biological replicates.

Protein expression was quantified using data-independent acquisition (DIA) LC–MS/MS. Processed Spectronaut output with cross-run median normalization and global imputation was used for all downstream analyses, while data completeness was evaluated in this section. Across all time points, 9,722 protein groups were identified with imputation, and an average of 8,939 protein groups per sample were detected without imputation (Fig. 8a). Among these, 7,494 protein groups were consistently identified in every sample, with an average missing value rate of 8% (Fig. 8b). Moreover, PCA revealed a clear separation of samples along the differentiation trajectory, with biological replicates clustering closely, indicating high reproducibility and consistent temporal progression in the dataset (Fig. 8c).

## Data Availability

RNA-seq data were deposited in the NCBI GEO database under accession number GSE305933^16^ (https://identifiers.org/geo:GSE305933). Ribosome profiling data were deposited in the NCBI GEO database under accession number GSE305934^17^ (https://identifiers.org/geo:GSE305934). Mass spectrometry data were deposited in the MassIVE repository under accession number MSV000098914^18^ (10.25345/C5NP1WX5Z). Supplementary annotation file has been deposited in Figshare^19^ (10.6084/m9.figshare.30757940), providing the BED file used for various downstream analyses.

## Code availability

Pre-processing scripts for RNA-seq and ribosome profiling datasets are available in the GitHub repository (https://github.com/ak3952/scientific_data_hESCtoPHcellDifferentiation).

## References

[1] Thomson, J. A. Embryonic stem cell lines derived from human blastocysts. *Science (1979)* **282**, 1145–1147, 10.1126/science.282.5391.1145 (1998). 10.1126/science.282.5391.1145 9804556

[2] Keskin, A. *et al*. Temporal multiomics gene expression data of human embryonic stem cell-derived cardiomyocyte differentiation. *Scientific Data* **12**, 1–13, 10.1038/s41597-025-05655-9 (2025). 40721591 10.1038/s41597-025-05655-9 PMC12304179

[3] Vogel, C. & Marcotte, E. M. Insights into the regulation of protein abundance from proteomic and transcriptomic analyses. *Nat Rev Genet* **13**, 227–232, 10.1038/nrg3185 (2012). 22411467 10.1038/nrg3185 PMC3654667

[4] Vogel, C. *et al*. Sequence signatures and mRNA concentration can explain two-thirds of protein abundance variation in a human cell line. *Mol Syst Biol* **6**, 10.1038/msb.2010.59 (2010). 10.1038/msb.2010.59 PMC2947365 20739923

[5] Schwanhüusser, B. *et al*. Global quantification of mammalian gene expression control. *Nature* **473**, 337–342, 10.1038/nature10098 (2011). 21593866 10.1038/nature10098

[6] Korytnikov, R. & Nostro, M. C. Generation of polyhormonal and multipotent pancreatic progenitor lineages from human pluripotent stem cells. *Methods* **101**, 56–64, 10.1016/j.ymeth.2015.10.017 (2016). 26515645 10.1016/j.ymeth.2015.10.017

[7] Ingolia, N. T., Ghaemmaghami, S., Newman, J. R. S. & Weissman, J. S. Genome-wide analysis *in vivo* of translation with nucleotide resolution using ribosome profiling. *Science* **324**, 218–223, 10.1126/science.1168978 (2009). 19213877 10.1126/science.1168978 PMC2746483

[8] Ingolia, N. T., Brar, G. A., Rouskin, S., McGeachy, A. M. & Weissman, J. S. The ribosome profiling strategy for monitoring translation *in vivo* by deep sequencing of ribosome-protected mRNA fragments. *Nature Protocols* **7**, 1534–1550, 10.1038/nprot.2012.086 (2012). 22836135 10.1038/nprot.2012.086 PMC3535016

[9] Hornstein, N. *et al*. Ligation-free ribosome profiling of cell type-specific translation in the brain. *Genome Biol* **17**, 1–15, 10.1186/s13059-016-1005-1 (2016). 27380875 10.1186/s13059-016-1005-1 PMC4934013

[10] Rappsilber, J., Mann, M. & Ishihama, Y. Protocol for micro-purification, enrichment, pre-fractionation and storage of peptides for proteomics using StageTips. *Nature Protocols* **2**, 1896–1906, 10.1038/nprot.2007.261 (2007). 17703201 10.1038/nprot.2007.261

[11] Dobin, A. *et al*. STAR: ultrafast universal RNA-seq aligner. *Bioinformatics* **29**, 15–21, 10.1093/bioinformatics/bts635 (2013). 23104886 10.1093/bioinformatics/bts635 PMC3530905

[12] Love, M. I., Huber, W. & Anders, S. Moderated estimation of fold change and dispersion for RNA-seq data with DESeq2. *Genome Biol* **15**, 1–21, 10.1186/s13059-014-0550-8 (2014). 10.1186/s13059-014-0550-8 PMC4302049 25516281

[13] Liao, Y., Wang, J., Jaehnig, E. J., Shi, Z. & Zhang, B. WebGestalt 2019: gene set analysis toolkit with revamped UIs and APIs. *Nucleic Acids Res* **47**, W199–W205, 10.1093/nar/gkz401 (2019). 31114916 10.1093/nar/gkz401 PMC6602449

[14] Subramanian, A. *et al*. Gene set enrichment analysis: A knowledge-based approach for interpreting genome-wide expression profiles. *Proc Natl Acad Sci USA* **102**, 15545–15550, 10.1073/pnas.0506580102 (2005). 16199517 10.1073/pnas.0506580102 PMC1239896

[15] Ritchie, M. E. *et al*. limma powers differential expression analyses for RNA-sequencing and microarray studies. *Nucleic Acids Res* **43**, e47–e47, 10.1093/nar/gkv007 (2015). 25605792 10.1093/nar/gkv007 PMC4402510

[16] Keskin, A. *et al*. Temporal Multiomics Gene Expression Data across Human Embryonic Stem Cell-Derived Polyhormonal Cell Differentiation. *NCBI Gene Expression Omnibus (GEO)* https://identifiers.org/geo/GSE305933 (2025). 10.1038/s41597-026-06606-8 PMC12920657 41545424

[17] Keskin, A. *et al*. Temporal Multiomics Gene Expression Data across Human Embryonic Stem Cell-Derived Polyhormonal Cell Differentiation. *NCBI Gene Expression Omnibus (GEO)* https://identifiers.org/geo/GSE305934 (2025). 10.1038/s41597-026-06606-8 PMC12920657 41545424

[18] Keskin, A. *et al*. Temporal Multiomics Gene Expression Data across Human Embryonic Stem Cell-Derived Polyhormonal Cell Differentiation. *MassIVE*10.25345/C5NP1WX5Z (2025). 10.1038/s41597-026-06606-8 PMC12920657 41545424

[19] Keskin, A. *et al*. Temporal Multiomics Gene Expression Data across Human Embryonic Stem Cell-Derived Polyhormonal Cell Differentiation. *Figshare*10.6084/m9.figshare.30757940 (2025). 10.1038/s41597-026-06606-8 PMC12920657 41545424

[20] Babraham Bioinformatics - FastQC A Quality Control tool for High Throughput Sequence Data. https://www.bioinformatics.babraham.ac.uk/projects/fastqc/.

[21] Ewels, P., Magnusson, M., Lundin, S. & Käller, M. MultiQC: summarize analysis results for multiple tools and samples in a single report. *Bioinformatics* **32**, 3047–3048, 10.1093/bioinformatics/btw354 (2016). 27312411 10.1093/bioinformatics/btw354 PMC5039924

[22] Lauria, F. *et al*. riboWaltz: Optimization of ribosome P-site positioning in ribosome profiling data. *PLoS Comput Biol* **14**, e1006169, 10.1371/journal.pcbi.1006169 (2018). 30102689 10.1371/journal.pcbi.1006169 PMC6112680

